# Stagnating blood flow related to thrombus formation in pulmonary vein stump after left upper lobectomy

**DOI:** 10.1007/s11748-023-01926-7

**Published:** 2023-03-20

**Authors:** Takahito Nakano, Hiroyuki Kaneda, Tomohiro Murakawa

**Affiliations:** Department of Thoracic Surgery, Kansai Medical University, 2-5-1 Shin-Machi, Hirakata, Osaka 573-1010 Japan

**Keywords:** Blood flow stagnation, Thrombus, Pulmonary vein, Left upper lobectomy, Computational fluid dynamics

## Abstract

**Objectives:**

A thrombus can occur in the stump of the pulmonary vein after left upper lobectomy, potentially causing postoperative cerebral infarction. This study aimed to verify the hypothesis that stagnation of blood flow inside the pulmonary vein stump causes thrombus formation.

**Methods:**

The three-dimensional geometry of the pulmonary vein stump after left upper lobectomy was recreated using contrast-enhanced computed tomography. Blood flow velocity and wall shear stress (WSS) inside the pulmonary vein stump were analysed using the computational fluid dynamics (CFD) method and compared between the two groups (those with or without thrombus).

**Results:**

The volumes of average flow velocity per heartbeat < 10 mm/s, 3 mm/s, 1 mm/s (p-values 0.0096, 0.0016, 0.0014, respectively) and the volumes where flow velocity was always below the three cut-off values (p-values 0.019, 0.015, 0.017, respectively) were significantly larger in patients with a thrombus than in those without thrombus. The areas of average WSS per heartbeat < 0.1 Pa, 0.03 Pa, 0.01 Pa (p-values 0.0002, < 0.0001, 0.0002, respectively), and the areas where WSS was always below the three cut-off values (p-values 0.0088, 0.0041, 0.0014, respectively) were significantly larger in patients with thrombus than in those without thrombus.

**Conclusions:**

The area of blood flow stagnation in the stump calculated by CFD method was significantly larger in patients with than in those without thrombus. This result elucidates that stagnation of blood flow promotes thrombus formation in the pulmonary vein stump in patients who undergo left upper lobectomy.

**Supplementary Information:**

The online version contains supplementary material available at 10.1007/s11748-023-01926-7.

## Introduction

Lung cancer is the leading cause of cancer mortality worldwide, and surgery for lung cancer is widely performed with curative intent. Cerebral infarction can occur after surgery for lung cancer. According to a recent survey reported from Japan in 2018, the incidence of cerebral infarction after lung cancer surgery was 0.868% [1].

An association among certain pulmonary resection procedures, left upper lobectomy, and cerebral infarction was reported in 2012 [2]. In that report, cerebral infarction after left upper lobectomy was presumed to be caused by a thrombus in the stump of the pulmonary vein. Since then, several cerebral infarction cases after left upper lobectomy with a thrombus in the pulmonary vein stump, including our case experience, were reported [3–8]. Subsequent studies using Japan's National Clinical Database revealed that cerebral infarction after lung cancer was more common after left upper lobectomy than other types of pulmonary resections [9].

Based on data reported in 2013, the pulmonary vein stump after left upper lobectomy remains longer than that after other types of lobectomies. A mechanism was speculated and it was suggested that a thrombus is formed due to stagnation of blood flow inside a long pulmonary vein stump [10, 11]. In our previous study, thrombi in the pulmonary vein stump were observed in all patients after left upper lobectomy using our conventional techniques for pulmonary vein transection [12]. Given that thrombus formation in the early postoperative period of left upper lobectomy is not a rare event, we developed a surgical technique to shorten the pulmonary vein stump [12]. This surgical procedure significantly shortened the pulmonary vein stump length. It significantly reduced the incidence of thrombi in the pulmonary vein, although the relationship between blood flow stagnation and thrombus formation is still unknown.

The computational fluid dynamics (CFD) method has been performed in several fields to clarify the relationship between blood flow stagnation and thrombus formation, including rupture risk assessment after coil embolization of cerebral aneurysms and graft patency after revascularization of large vessels [13, 14].

This study analysed blood flow using the CFD method, focusing inside the pulmonary vein stump after left upper lobectomy, and verified the hypothesis that blood flow stagnation causes thrombus formation inside the pulmonary vein stump.

## Subjects

This study was approved by The Institutional Review Board (approval date: June 22, 2021; approval number: 2021055). The requirement for obtaining informed consent was waived because of the study’s retrospective nature.

The inclusion criteria were as follows: patients who underwent left upper lobectomy for lung cancer from July 2016 to September 2018, those who underwent contrast-enhanced computed tomography (CT) within 1 week after surgery, those who had no history of preoperative anticoagulant medication, and those who had no anatomical abnormalities, such as common trunk of the pulmonary veins. Patients who had a cerebral infarction after left upper lobectomy and a thrombus in the pulmonary vein stump were also included in this study.

## Methods

### Surgical techniques to dissect and ligate pulmonary vein

Left upper lobectomy plus lymphadenectomy was performed by three-port thoracoscopic surgery or thoracotomy. The pulmonary artery, pulmonary vein, and bronchi were transected with a stapler. The surgical technique for pulmonary vein dissection was previously reported [12]. Until March 2017, the pulmonary vein was dissected and transected without paying special attention to the length of the stump. In the new method, the pericardium on the central side of the pulmonary vein was peeled off first. Then the pulmonary vein was ligated with a silk thread at the central side, and the peripheral side was transected with a stapler.

### Blood flow analysis in the pulmonary vein stump

**Fig. 1:**
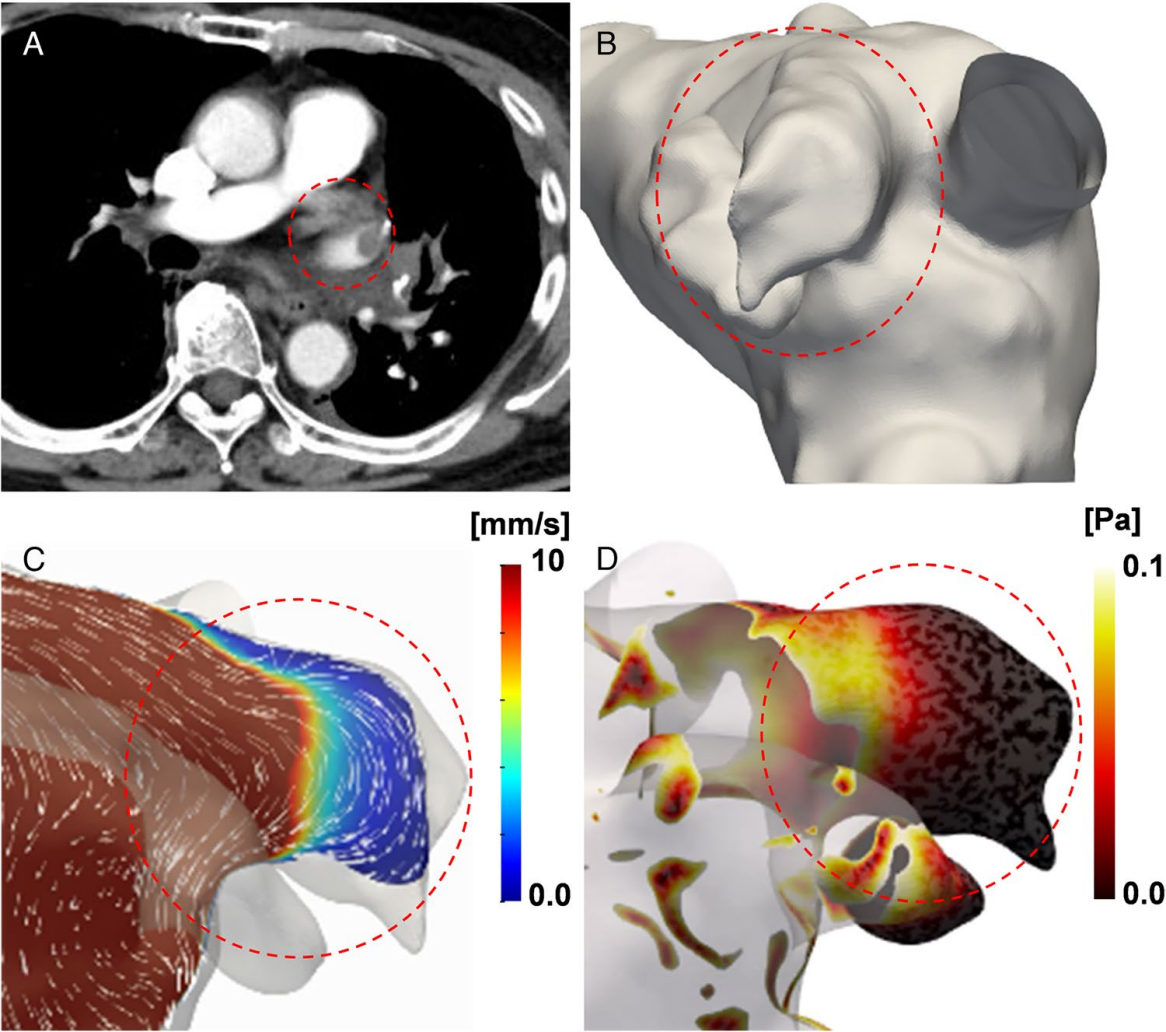
**A** Contrast-enhanced computed tomography was performed within 1 week after surgery. In this case, a thrombus was detected in the pulmonary vein stump (circle). **B** Image data in a Digital Imaging and Communications in Medicine format were transferred into three-dimensional patient-specific geometries, and the pulmonary vein stump was reconstructed (circle). **C** The computational fluid dynamics (CFD) method analysed the blood flow velocity vector. In this case, the low-velocity area presented in blue is shown in the pulmonary vein stump (circle). **D** The CFD method analysed the wall shear stress (WSS). In this case, low WSS area presented with dark red is shown in the pulmonary vein stump (circle)

The details of CFD method are based on those of previous studies [15–17]. The vessel geometry was created from enhanced multidetector-row CT (Fig. 1A) using ZioStation (ZioSoft, Tokyo, Japan) and Blender (Blender Foundation, Amsterdam, the Netherlands) (Fig. 1B). In cases in which a thrombus was detected, the area of interest was traced inside the pulmonary vein stump regardless of the thrombus. Computational meshes were created with ANSYS-ICEM CFD 16.0 (ANSYS Japan, Tokyo, Japan). Flow rate inlet boundary conditions were set for each pulmonary vein. The flow rates in one cardiac cycle of the pulmonary vein were measured using 4D flow magnetic resonance imaging in healthy volunteers and corrected to 5L/min in total.

The outlet boundary conditions for the left atrium were set with pressure boundary conditions [18]. The finite volume method was used to solve Navier–Stokes equation with the convergence criteria of 1.0E-5 for all parameters on the open source CFD software OpenFOAM (Open FOAM Foundation). Unified heartrate, left atrial outlet pressure, blood density, and viscosity coefficient values were used across all subjects for analysis (heart rate 60 beats/min, left atrial outlet pressure 16.0 mmHg, blood density 1060 kg/m^3^, viscosity coefficient 0.004 Pa*s).

As an index for evaluating stagnation of blood flow in the stump, we calculated blood flow velocity (Fig. 1C) and wall shear stress (WSS) (Fig. 1D). WSS is a vector with force and direction to the vessel wall at a certain point in time [19].

### Statistical analysis

We statistically compared the patients’ background data and results of blood flow analysis within the pulmonary vein stump between the two groups (thrombus and non-thrombus). Since this was an exploratory study, we set three arbitrary cut-off values: 10 mm/s, 5 mm/s, and 3 mm/s for flow velocity and 0.1 Pa, 0.03 Pa, and 0.01 Pa for WSS. Values were set in two ways: the volume or area where the average value during a heartbeat is less than the cut-off value and the volume or area where the average value is always less than the cut-off value. Continuous variables are presented as mean and categorical variables as the number of patients. Statistical analysis was performed to investigate the risk factors for thrombus formation. Student *t*-test and Fisher’s exact test were used to compare continuous and categorical data, respectively. The association between the continuous variables was analyzed using the Pearson’s correlation tests. A p-value < 0.05 was considered statistically significant. All statistical analyses were performed using JMP software version 13.2.1 (SAS Institute, Inc., Cary, NC, USA).

## Results

This study included a total of 15 patients. The clinical background data of these 15 patients are shown in Table 1. There were six cases with thrombus and nine without thrombus. No significant differences were noted between the thrombus and non-thrombus groups regarding age, sex, and preoperative and postoperative blood parameters. The details of the six cases with thrombus are shown in Table 2. Postoperative cerebral infarction occurred in one of the six patients, and the details were previously reported [8]. In the remaining five patients, thrombus in the pulmonary vein stump was detected with no symptoms, and anticoagulant therapy was administered, resulting in the disappearance of the thrombus in the follow-up contrast-enhanced CT without thrombosis.

**Table 1:** Patient characteristics of groups with or without thrombus in the pulmonary vein stump

	Patients with thrombus (n = 6)	Patients without thrombus (n = 9)	p-value
Age (years, mean)	69.7	69.0	0.90
Sex, (male/female)	5/1	5/4	0.58
Previous episode of CI (yes/no)	0/6	1/8	> .99
Preoperative haematological parameters			
HTC (%, mean)	41.6	39.3	0.18
WBC count (10^4^/mm^3^, mean)	73.8	65.6	0.33
Platelet count (10^4^/mm^3^, mean)	23.9	25.9	0.60
PT-INR (mean)	1.02	1.03	0.81
APTT (mean)	31.6	29.2	0.30
Surgical procedure			
VATS/Open (n)	1/5	2/7	1.0
PV ligation (yes/no)	2/4	9/0	0.011
PV stump length (mm, mean)	18.5	5.4	< .001
Postoperative day 1 haematological parameters			
HTC (%, mean)	35.5	32.6	0.27
WBC count (10^4^/mm^3^, mean)	113.2	98.3	0.26
Platelet count (10^4^/mm^3^, mean)	23.0	21.7	0.71
Postoperative CI within 3 months (n)	1	0	0.40

*APTT* activated partial thromboplastin time, *CI* cerebral infarction, *HTC* haematocrit, *PT-INR* prothrombin time-international normalized ratio, *PV* pulmonary vein, *VATS* video-assisted thoracic surgery, *WBC* white blood cell

**Table 2:** Clinical characteristics of the six patients with a thrombus in the pulmonary vein stump

Case No	1	2	3	4	5	6
Sex (M/F)	77	81	66	69	50	75
VATS/Open	Open	VATS	VATS	VATS	VATS	VATS
PV ligation (yes/no)	No	No	No	No	Yes	Yes
Stump length (mm)	13.5	17.2	18.0	27.6	14.6	20.1
Postoperative CI within 3 months (yes/no)	Yes	No	No	No	No	No
Area of average blood flow velocity (cm^3^)						
< 10 mm/s	2.1	1.1	1.9	2.9	0.8	2.4
< 5 mm/s	1.4	0.7	1.6	2.5	0.7	2.2
< 3 mm/s	1.0	0.5	1.2	2.2	0.6	2.0
Area of all time blood flow velocity (cm^3^)						
< 10 mm/s	1.3	0.1	0.4	2.5	0.5	2.2
< 5 mm/s	0.7	0.1	0.2	2.0	0.4	1.9
< 3 mm/s	0.4	0.0	0.2	1.7	0.3	1.6
Area of average WSS (cm^2^)						
< 0.1 Pa	8.2	6.0	7.5	9.4	4.7	8.6
< 0.03 Pa	5.8	3.3	5.7	8.7	4.1	7.9
< 0.01 Pa	2.7	1.7	3.7	7.3	3.2	6.7
Area of all time WSS (cm^2^)						
< 0.1 Pa	6.9	0.9	3.7	9.1	3.1	8.2
< 0.03 Pa	3.1	0.3	1.1	7.0	2.3	7.1
< 0.01 Pa	1.2	0.1	0.5	5.5	1.1	5.3

*M* male, *F* female, *VATS* video-assisted thoracic surgery, *PV* pulmonary vein, *CI* cerebral infarction, *WSS* wall shear stress

**Fig. 2:**
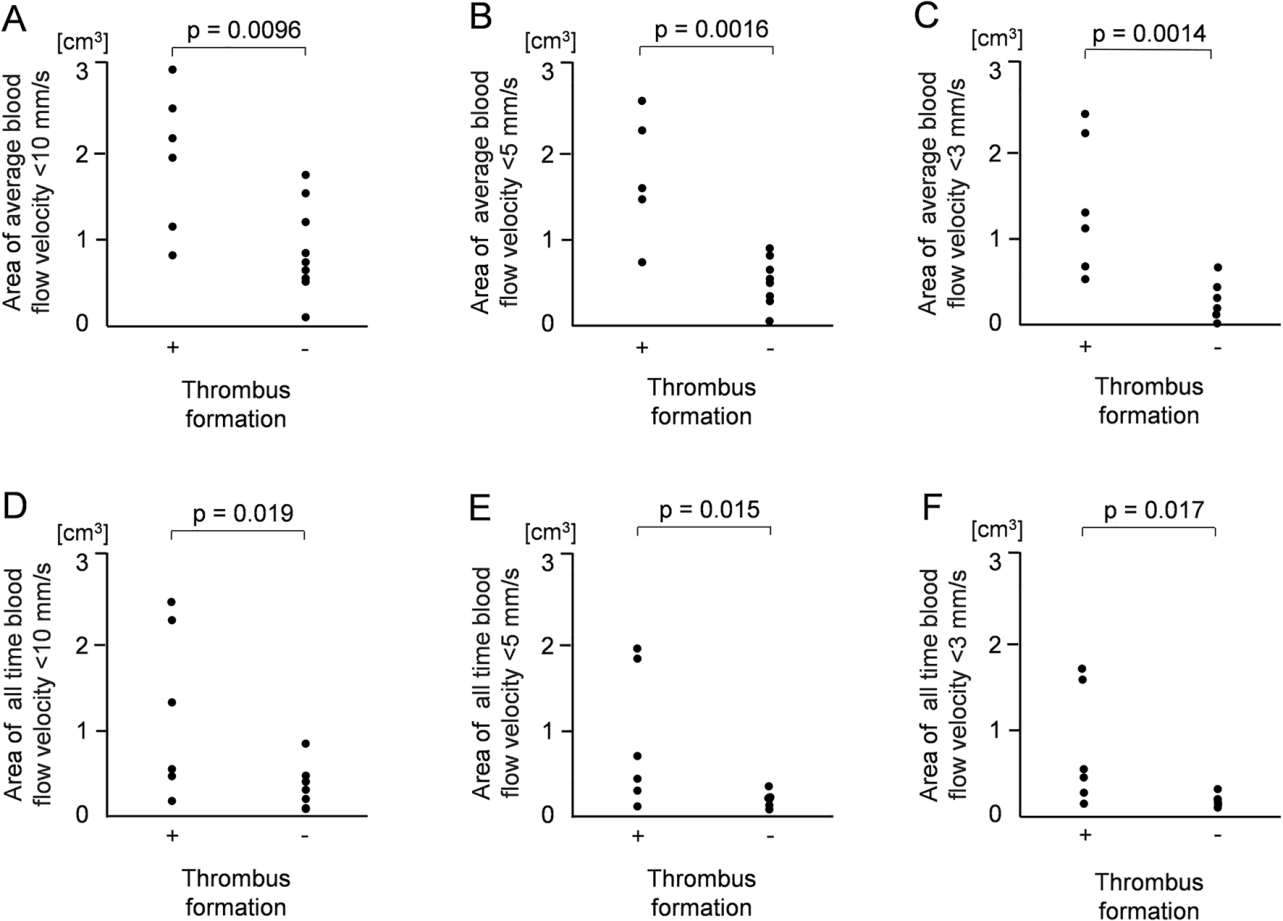
Comparison of low blood flow velocity volumes between the two (thrombus and non-thrombus) groups. **A**, **B**, **C** For the three cut-off values of 10 mm/s, 5 mm/s, and 3 mm/s, statistically significant differences were noted between the two groups in the volume where an average blood flow velocity per heartbeat was less than the cut-off value (p-values 0.0096, 0.0016, 0.0014, respectively). **D**, **E**, **F** For the three cut-off values of 10 mm/s, 5 mm/s, and 3 mm/s, statistically significant differences were noted between the two groups in the volume where the blood flow velocity was always below the cut-off value (p-values 0.019, 0.015, 0.017, respectively)

**Fig. 3:**
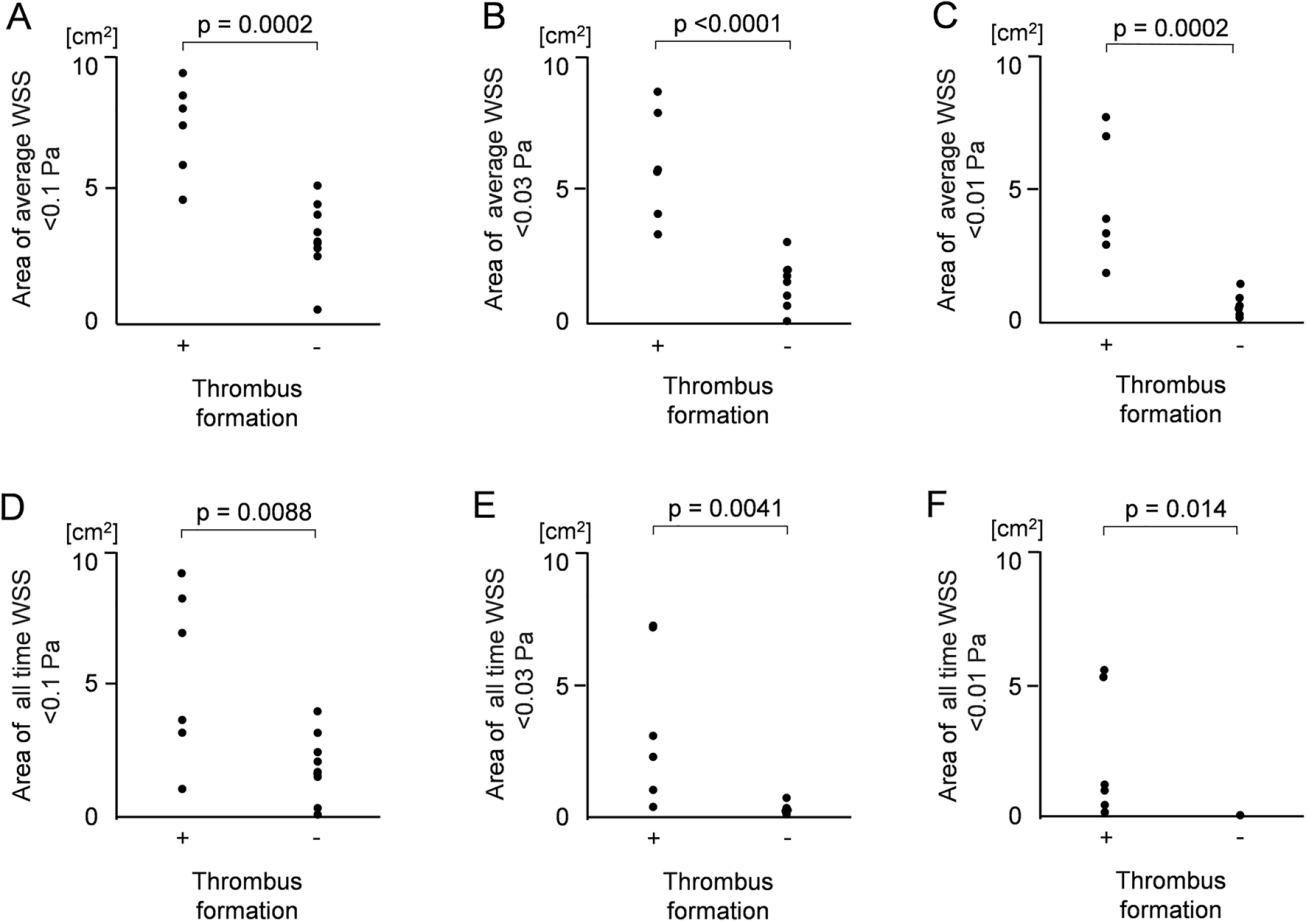
Comparison of low wall shear stress (WSS) areas between the two (thrombus and non-thrombus) groups. **A**, **B**, **C** For the three cut-off values of 0.1 Pa, 0.03 Pa, and 0.01 Pa, statistically significant differences were noted between the two groups in the area where an average WSS per heartbeat was less than the cut-off value (p-values 0.0002, < 0.0001, 0.0002, respectively). For the three cut-off values of 0.1 Pa, 0.03 Pa, and 0.01 Pa, statistically significant differences were noted between the two groups in the area where the WSS was always less than the cut-off value (p-values 0.0088, 0.0041, 0.0014, respectively)

A comparison of blood flow velocity inside the pulmonary vein stump between the thrombus and non-thrombus groups is shown in Fig. 2. The mean volumes of an average blood flow velocity < 10 mm/s were 1.87 cm^3^ and 0.84 cm^3^ in the thrombus and non-thrombus groups, respectively (Fig. 2A). The mean volumes of an average blood flow velocity < 5 mm/s were 1.51 cm^3^ and 0.44 cm^3^ in the thrombus and non-thrombus groups, respectively (Fig. 2B). The mean volumes of an average blood flow velocity < 3 mm/s were 1.25 cm^3^ and 0.26 cm^3^ in the thrombus and non-thrombus groups, respectively (Fig. 2C). For the three cut-off values of 10 mm/s, 5 mm/s, and 3 mm/s, statistically significant differences were noted between the two groups in the volumes where the average blood flow velocity per heartbeat was less than the cut-off value (p-values 0.0096, 0.0016, 0.0014, respectively). The mean volume where the blood flow velocity was consistently < 10 mm/s was 1.17 cm^3^ and 0.27 cm^3^ in the thrombus and non-thrombus groups, respectively (Fig. 2D). The mean volume where the blood flow velocity was always < 5 mm/s was 0.86 cm^3^ and 0.09 cm^3^ in the thrombus and non-thrombus groups, respectively (Fig. 3E). The mean volume where the blood flow velocity was always < 3 mm/s was 0.69 cm^3^ and 0.05 cm^3^ in the thrombus and non-thrombus groups, respectively (Fig. 3F). For the three cut-off values of 10 mm/s, 5 mm/s, and 3 mm/s, statistically significant differences were noted between the two groups in the volume of the region where the blood flow velocity was always below the cut-off value (p-values 0.019, 0.015, 0.017, respectively).

A comparison of WSS inside the pulmonary vein stump between the two groups is shown in Fig. 3. The mean areas of an average WSS < than 0.1 Pa were 7.39 cm^2^ and 3.24 cm^2^ in the thrombus and non-thrombus groups, respectively (Fig. 3A). The mean areas of an average WSS < 0.03 Pa were 5.03 cm^2^ and 1.51 cm^2^ in the thrombus and non-thrombus groups, respectively (Fig. 3B). The mean areas of an average WSS < 0.01 Pa were 4.21 cm^2^ and 0.39 cm^2^ in the thrombus and non-thrombus groups, respectively (Fig. 3C). For the three cut-off values of 0.1 Pa, 0.03 Pa, and 0.01 Pa, statistically significant differences were noted between the two groups in the area where an average WSS per heartbeat was less than the cut-off value (p-values 0.0002, < 0.0001, 0.0002, respectively). The mean areas where the WSS was always < 0.1 Pa were 5.33 cm^2^ and 1.64 cm^2^ in the thrombus and non-thrombus groups, respectively (Fig. 3D). The mean areas where the WSS was always < 0.03 Pa were 3.49 cm^2^ and 0.13 cm^2^ in the thrombus and non-thrombus groups, respectively (Fig. 3E). The mean areas where the WSS was always < 0.01 Pa were 2.29 cm^2^ and 0.004 cm^2^ in the thrombus and non-thrombus groups, respectively (Fig. 3F). For the three cut-off values of 0.1 Pa, 0.03 Pa, and 0.01 Pa, statistically significant differences were noted between the two groups in the area where WSS was always below the cut-off value (p-values 0.0088, 0.0041, 0.0014, respectively).

**Fig. 4:**
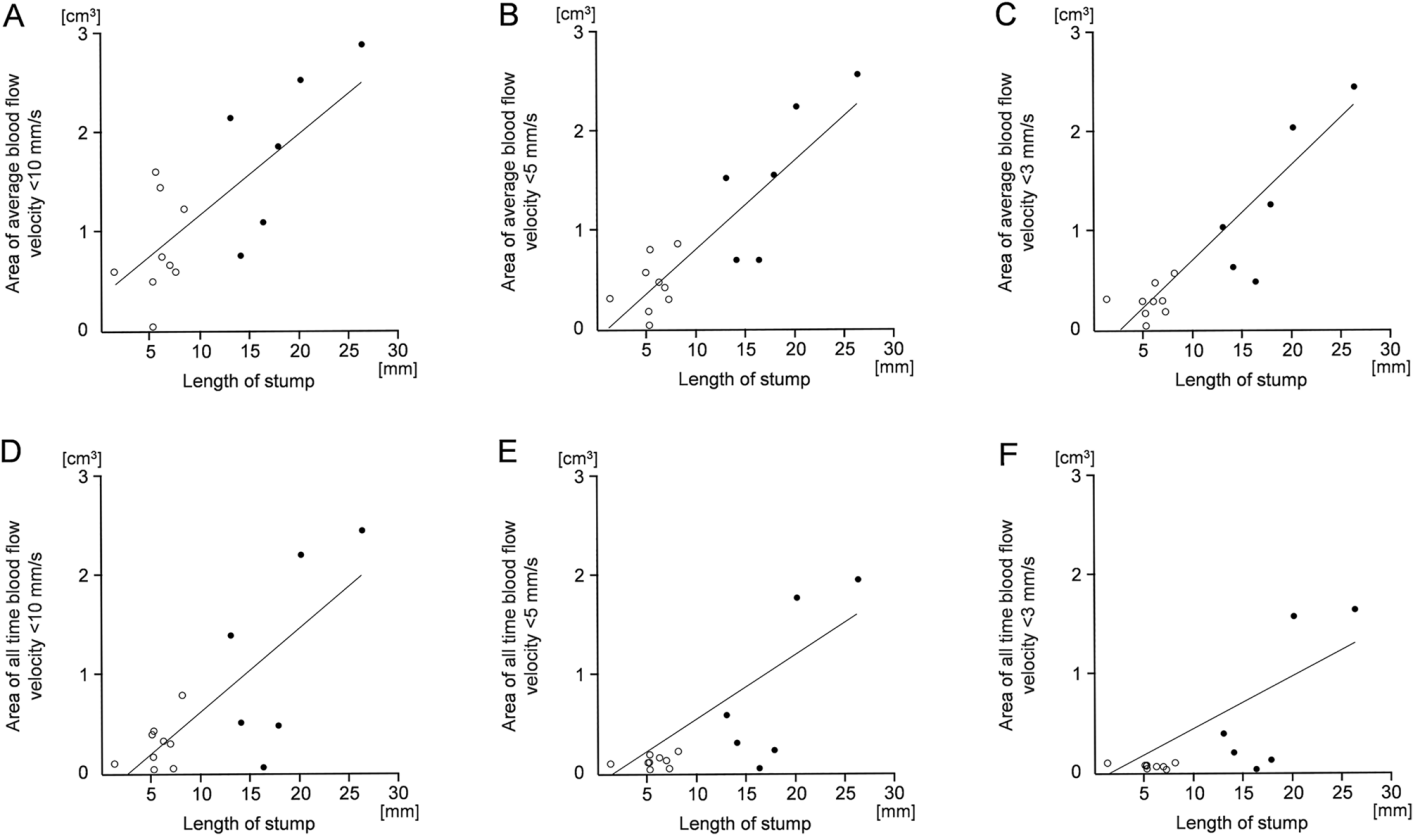
The relation between the length of the pulmonary vein stump and the volume where the blood flow velocity was always low. The black and white dots in each panel represent the cases with and without thrombus, respectively. **A**, **B**, **C** For the cut-off values of 10 mm/s, 5 mm/s, and 3 mm/s, respectively, statistically significant relationships were noted between the length of stump and the volume where the blood flow velocity per heartbeat was always less than the cut-off value (p-values < 0.0001, < 0.0001, < 0.0001, respectively). **D**, **E**, **F** For the cut-off values of 10 mm/s, 5 mm/s, and 3 mm/s, respectively, statistically significant relationships were noted between the length of stump and the volume where the blood flow velocity was always below the cut-off value (p-values 0.0008, 0.0004, 0.0004, respectively)

The relation between the length of the pulmonary vein stump and blood flow velocity is shown in Fig. 4. The volume where the blood flow velocity was always < 10 mm/s (Fig. 4A), < 5 mm/s (Fig. 4B), and < 3 mm/s (Fig. 4C) per heartbeat represented the correct proportions of the length of the pulmonary vein with statistical significance (p-values 0.0010, < 0.0001, < 0.0001, respectively). The volume where the blood flow velocity was always < 10 mm/s (Fig. 4D), < 5 mm/s (Fig. 4E), and < 3 mm/s (Fig. 4F) represented the correct proportions of the length of the pulmonary vein with statistical significance (p-values 0.0008, 0.0004, 0.0004, respectively).

**Fig. 5:**
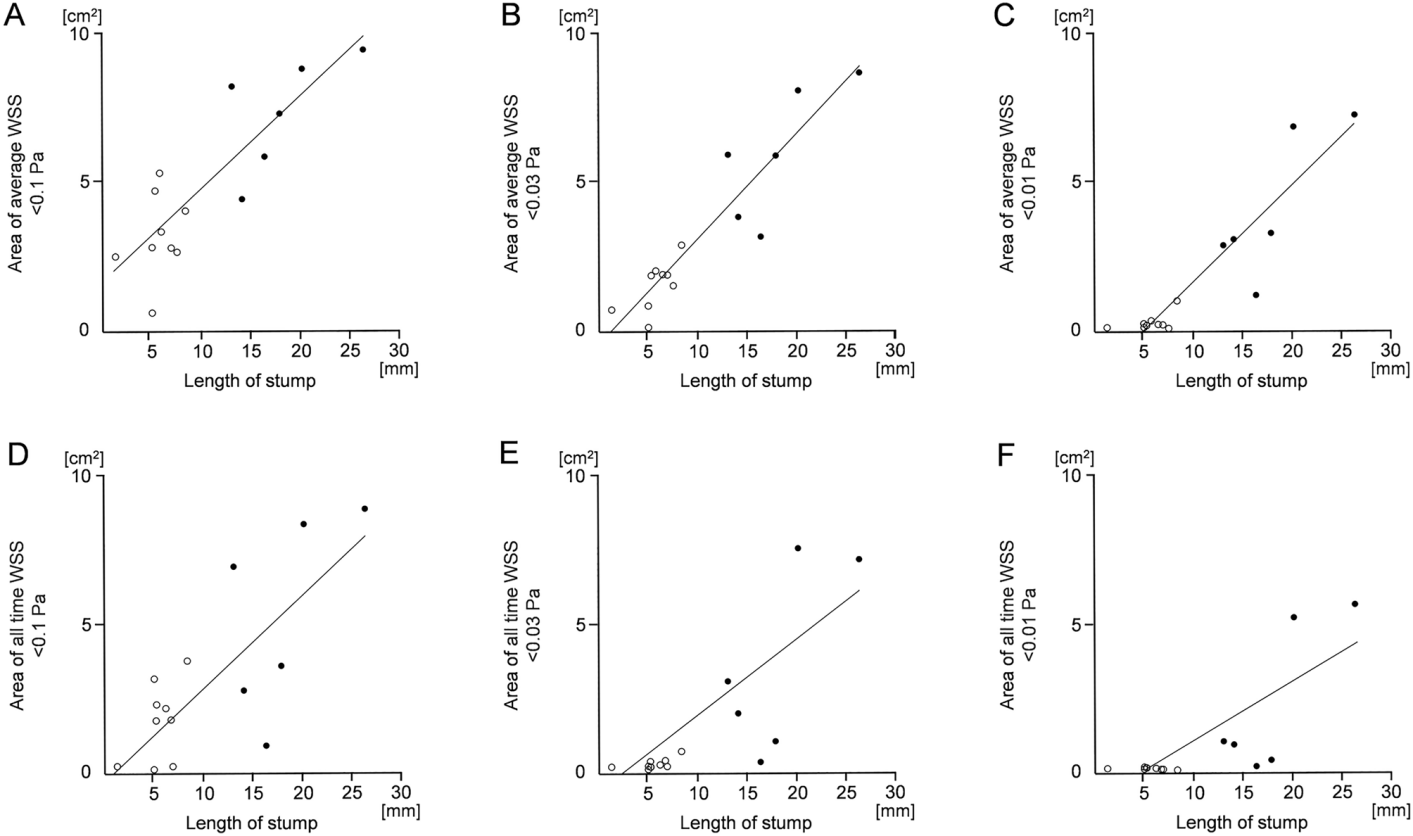
The relation between the length of the pulmonary vein stump and the area of low wall shear stress (WSS). The black and white dots in each panel represent the cases with and without thrombus, respectively. **A**, **B**, **C** For the cut-off values of 0.1 Pa, 0.03 Pa, and 0.01 Pa, respectively, statistically significant relationships were noted between the length of the stump and the area where the average WSS per heartbeat was less than the cut-off value (p-values 0.0010, < 0.0001, < 0.0001, respectively). **D**, **E**, **F** For the cut-off values of 0.1 Pa, 0.03 Pa, and 0.01 Pa, respectively, significant relationships were noted between the length of the stump and the area where the WSS was always less than the cut-off value (p-values 0.0009, 0.0002, 0.0003, respectively)

The relation between the length of the pulmonary vein stump and WSS is shown in Fig. 5. The areas with average WSS < 0.1 Pa (Fig. 4A), < 0.03 Pa (Fig. 4B), and < 0.01 Pa (Fig. 4C) per heartbeat represented the correct proportions of the length of the pulmonary vein with statistical significance (p-values < 0.0001, < 0.0001, < 0.0001, respectively). The areas where the WSS was always < 0.1 Pa (Fig. 4D), < 0.03 Pa (Fig. 4E), and < 0.01 Pa (Fig. 4F) represented the correct proportions of the length of the pulmonary vein with statistical significance (p-values 0.0009, 0.0002, 0.0003, respectively).

## Discussion

In this study, we examined blood flow inside the pulmonary vein stump using the CFD method in patients who underwent left upper lobectomy and revealed that blood flow stagnation inside the pulmonary vein stump was related to thrombus formation. These results elucidate that stagnation of blood flow promotes thrombus formation in the pulmonary vein stump in patients who undergo left upper lobectomy. This report is the first to clearly show the mechanism of thrombus formation in the pulmonary vein stump, allowing suggestions for preventing thrombus formation.

Blood flow stagnation was presumed to be an important factor in causing thrombus formation and subsequent thrombosis, such as cerebral infarction, based on the study by Ohtaka et al. in 2013 [10]. Although this study reported that the pulmonary vein stump after left upper lobectomy was longer than that after other types of lobectomies, and that thrombi within the stump were found only in patients who underwent left upper lobectomy, blood stagnation in the pulmonary vein stump has not been visualized to date. There are previous reports related to blood flow analysis of thrombosis of the pulmonary vein stumps. Nakaza et al. reported that turbulence in the left atrium occurred after left upper lobe resection [20], and Umehara et al. reported that blood flow stagnation occurred in an area of the left atrium near the pulmonary vein stump [21, 22]. In these studies, blood flow in the left atrium or near the pulmonary vein stump after left upper lobectomy differed from other lobectomies. However, blood flow inside the pulmonary vein stump had not been examined. In our study, the CFD method reconstructed blood flow inside the pulmonary vein stump where a thrombus can form. The stagnation, represented as volume of low blood flow velocity or area of low WSS, was significantly larger in the patients with thrombus than in patients without thrombus. Our analysis results match the speculated mechanism that the thrombus is formed by the stagnation of blood flow inside the pulmonary vein stump [10, 11].

We have performed surgical procedures to shorten the pulmonary vein stump to prevent thrombosis in the case of the left upper lobe resection and have confirmed its effectiveness [12]. Our ligation method does not require complicated handwork. Both ligation of the central side and subsequent stapling of the peripheral side of the pulmonary vein can be safely performed without the risk of injuring the mediastinal structure. Moreover, our ligation method is feasible both in open and thoracoscopic settings. In addition to the advantages previously described, the present study supports the validity of the ligation method with the aspect of blood stagnation.

Anticoagulant therapy is also an effective method of preventing thrombi and thrombosis. In our previous review, thrombus was confirmed to disappear with anticoagulant therapy in almost all patients, and an increased risk of subsequent embolism was not present.^8^ However, there is insufficient evidence regarding the optimal timing of anticoagulant therapy. We also observed recurrent stroke after administering anticoagulant therapy for pulmonary vein thrombosis [8]. Overall, the incidence of embolism is rarer than the incidence of thrombi detected in the pulmonary vein stump; therefore, whether early postoperative anticoagulant therapy outweighs the risks of complications such as postoperative bleeding is questionable. Considering these factors, we did not examine ROC with the CFD data to predict thrombus in the pulmonary vein stump.

The limitation was that we did not clarify all elements in the mechanism of thrombus formation. Contrast-enhanced CT was performed within one week of the surgery in our study. The period chosen in the present study appears feasible because the frequency of thrombus detected by contrast-enhanced CT appears to remain almost constant until 2 weeks after the surgery [23]. We performed contrast-enhanced CT only in cases with left upper lobectomy because it was specifically related to the thrombus or thrombosis compared to other lobectomies [1, 2]. Therefore, only cases with left upper lobectomy were included in the CFD analysis. Recent reports suggest that although the frequency is relatively low, lobectomies other than left upper lobectomy also cause thrombus formation [23]. However, further research is needed to confirm if blood flow stagnation induces thrombus formation in cases with a long pulmonary vein stump after other lobectomies. The elements of thrombophilia are shown as Virchow's triad: stasis of blood flow, hypercoagulability, and endothelial injury [24]. We focused on the stasis of blood flow, and as a result, we obtained outcomes that could explain the mechanism of thrombus formation after left upper lobe resection. However, different mechanisms of thrombus formation should be clarified for the following reasons. A long pulmonary vein stump does not always form a thrombus. Thrombus formation rarely results in an embolism. These facts suggest that various factors are involved in the process from thrombus formation to embolism development. Potential other factors, such as increased coagulation due to the pathology of cancer and the effect of pulmonary vein dissection on the vascular endothelium, will need to be examined in terms of hypercoagulability or endothelial injury. In addition, many patients have heart diseases such as valvular disease or atrial fibrillation, and it is speculated that heart diseases affect blood flow in the left atrium and pulmonary vein stump. In the present study, patients with a history of heart disease were excluded.

## Conclusion

This study analysed blood flow stagnation inside the pulmonary vein stump after left upper lobectomy for lung cancer. The area of ​​blood flow stagnation in the stump was significantly larger in patients with postoperative thrombus formation than in patients without postoperative thrombus formation. This result indicates that stagnation of blood flow promotes thrombus formation in patients who undergo left upper lobectomy in the pulmonary vein stump.

### Supplementary Information

Below is the link to the electronic supplementary material.

Supplementary file1 This video presents the dynamic changes of the velocity vector and wall shear stress (WSS) analysed by the computational fluid dynamics method. In case 1, a thrombus in the pulmonary vein stump was detected by postoperative contrast-enhanced computed tomography (CT). The low blood velocity presented with blue colour, and the low area of WSS presented with a dark red colour are shown inside the pulmonary vein stump. In case 2, postoperative contrast-enhanced CT did not detect a thrombus in the pulmonary vein stump. Almost all of the inside of the pulmonary vein stump has a velocity vector of > 10 mm/s (given in dark red) and WSS of > 0.1 Pa (given in white) (MPG 8996 KB)
